# NGF Signaling Interacts With the Hippo/YAP Pathway to Regulate Cervical Cancer Progression

**DOI:** 10.3389/fonc.2021.688794

**Published:** 2021-10-14

**Authors:** Lijun Wang, Jing Li, Rongli Wang, He Chen, Ruiqi Wang, Wei Wang, Xinyuan Yang

**Affiliations:** ^1^ Department of Obstetrics and Gynecology, the First Affiliated Hospital of Xi’an Jiaotong University, Xi’an, China; ^2^ Center for Translational Medicine, the First Affiliated Hospital of Xi’an Jiaotong University, Xi’an, China; ^3^ Department of Anesthesiology, the First Affiliated Hospital of Xi’an Jiaotong University, Xi’an, China

**Keywords:** cervical cancer, LATS1, NGF, progression, YAP

## Abstract

Nerve growth factor (NGF) is increasingly implicated in cervical cancer progression, but its mechanism in cervical cancer is unclear. Here, studies demonstrate that NGF inhibits the Hippo signaling pathway and activates Yes-associated protein (YAP) to induce cervical cancer cell proliferation and migration. Our results suggested that stimulation of NGF promoted cell growth and migration and activated YAP in HeLa and C-33A cell lines. The expression of YAP target genes (CTGF and ANKRD1) was upregulated after NGF treatment. The NGF inhibitor Ro 08-2750 and siRNA-mediated NGF receptor gene silencing suppressed HeLa and C-33A cells proliferation and migration, activated large suppressor kinase 1 (LATS1) kinase activity, and suppressed YAP function. In addition, the expression of YAP target genes (CTGF and ANKRD1) was suppressed by Ro 08-2750 treatment in HeLa and C-33A cells. Interestingly, proliferation was significantly higher in NGF-treated cells than in control cells, and this effect was completely reversed by the YAP small molecule inhibitor-verteporfin. Furthermore, the mouse xenograft model shows that NGF regulates YAP oncogenic activity *in vivo*. Mechanistically, NGF stimulation inactivates LATS1 and activates YAP, and NGF inhibition was found to induce large suppressor kinase 1 (LATS1) phosphorylation. Taken together, these data provide the first direct evidence of crosstalk between the NGF signaling and Hippo cancer pathways, an interaction that affects cervical cancer progression. Our study indicates that combined targeting of the NGF signaling and the Hippo pathway represents a novel therapeutic strategy for treatment of cervical cancer.

## Introduction

Cervical cancer is the leading cause of cancer-related mortality in women in developing countries (1). Despite a decrease in incidence and disease-specific mortality worldwide, 50% of cervical cancer patients in developing countries and over 10% in developed countries are diagnosed with late-stage disease (2). Therefore, the approach of novel diagnostic, prognostic, and therapeutic strategies for clinically advanced cervical cancers is warranted.

Nerve growth factor (NGF) is an important neuropeptide of the neurotrophin (NT) family, as a complex composed of three noncovalently linked subunits, α, β, and γ. The β subunit exhibits all the biological activities ascribed to NGF. Therefore, NGF generally refers to β-NGF (3). NGF interacts with two separate receptors: Tyrosine kinase receptor A (TrkA) and p75^NTR^ (4). TrkA is a high-affinity tyrosine kinase receptor that mainly mediates multiple effects of NGF signaling to promote survival, proliferation, and invasiveness of cells (5). P75^NTR^ is a low affinity, nonselective neurotrophin receptor, which lacks intrinsic catalytic activity. Originally, the attention of NGF/TrkA was mainly focused on nervous system development (6, 7). During recent years, accumulating data support a role for NGF/TrkA signaling in tumorigenesis and progression, including pancreatic cancer, breast cancer, and prostate cancer (8, 9). For example, in breast cancer, aberrant activation of TrkA leads to constitutive activation of the PI3K/AKT and Ras/MAPK pathways, resulting in tumor cell proliferation and invasion (10). The emerging data have shown that NGF/TrkA are overexpressed in cervical squamous cell carcinoma, but very low levels in normal tissues, which are correlated with the initiation, progression, and prognosis of cervical cancer (11). Information regarding the NGF/TrkA potential role in the progression of cervical cancer remains unclear.

The Hippo pathway is responsible for controlling organ size and growth by promoting apoptosis and limiting cell proliferation (12). The core part of the Hippo pathway consists of scaffold proteins and a kinase cascade and coactivators. In mammals, the core kinase of the Hippo pathway consists of STE20-like kinase 1/2 (Mst1/2), large suppressor kinase 1/2 (Lats1/2), and the RASSF family of proteins (13, 14). Activation of the Hippo pathway ultimately leads to the phosphorylation and subsequent cytosolic sequestration and/or degradation of the Hippo pathway transducers Yes-associated protein (YAP) and transcriptional coactivator with PDZ binding motif (TAZ) through a kinase cascade (15). In nucleus, YAP/TAZ interacts significantly with TEA DNA-binding proteins transcription factors and forms the YAP/TAZ-TEAD complex that mediates proliferative and prosurvival genes such as the connective tissue growth factor (CTGF), cysteine-rich angiogenic inducer 61 (CYR61), Ankyrin repeat domain 1 (ANKRD1), and others to promote cell survival, proliferation, and growth (16). YAP/TAZ dysregulation is associated with several types of cancer (17). A comprehensive survey also revealed YAP overexpression in most tumor types: lung, ovarian, pancreatic, colorectal, prostate, and hepatocellular carcinomas (18). YAP is the main effector molecule of the Hippo signaling pathway, which can regulate the expression of a large number of genes related to promoting cell proliferation and inhibiting cell apoptosis. The development of inhibitors targeting YAP is an important development in cancer treatment research targeting the Hippo signaling pathway. A study shows that YAP plays a central role in controlling the progression of cervical cancer, and YAP expression is associated with a poor prognosis for cervical cancer (19). Research reveals the overexpression of NGF in cervical squamous cell carcinoma (SCC) in the mean time (20). Hayakawa Y reveals that ablation of Dclk1^+^ cells or blockade of NGF/Trk signaling inhibited epithelial proliferation and tumorigenesis, in part through the suppression of YAP function within the gastrointestinal stem cell niche (21). More importantly, we performed a targeted kinase inhibitor screen in human cancer cells to identify novel Hippo pathway regulators in previous studies. We identified that the inhibitor of NGF decreased YAP-driven transcription, cancer cell proliferation, and migration (22). It can be seen that there is a close relationship between NGF/TrkA and YAP. Complete understanding of the molecular pathways controlling the Hippo pathway is critical for the development of novel Hippo pathway-specific therapeutics.

In this study, we found the role of the NGF signaling in cervical cancer and its relationship with the Hippo pathway. Since YAP is overexpressed in cervical cancer (19), we used HeLa (HPV positive) and C-33A (HPV negative) cervical cancer cell lines to investigate how the Hippo pathway is regulated in cervical cancer cells by measuring the protein expression and enzymatic activity of essential components of the pathway. The purpose of using two cervical cancer cell lines (Hela and C-33A) with different HPV status is to exclude the confounding factor influence of the HPV status on the Hippo pathway. Furthermore, tumor xenograft animal models were established to observe the important role of NGF *in vivo* and its regulatory mechanisms. Our results confirmed that NGF was critical for the proliferation and metastasis of cervical cancer cells and increases cervical cancer progression by inhibiting Hippo signaling. Taken together, NGF was important for YAP oncogenic activity. Our results provide the first direct evidence of NGF/TrkA crosslinking with Hippo signaling pathways in cervical cancer.

## Materials and Methods

### Cell Culture

HeLa cells were provided by Professor Xu Li (Center for Translational Medicine, the First Affiliated Hospital of Xi’an Jiaotong University). C-33A cells were obtained from Shanghai Genechem (Shanghai, China). Both cell lines were cultivated in Modified Eagle’s medium/EBSS medium (MEM/EBSS, HyClone, USA) supplemented with 10% fetal bovine serum (FBS), 100 units/ml penicillin, and 100 µg/ml streptomycin. Cells were cultured in a 5% CO_2_ humidified incubator at 37°C. Cells were not maintained in culture for longer than 3 months to ensure that the passage number remained fit for purpose.

### SiRNA Transfection

SiRNA duplex (ON-TARGET Plus SMARTPool) targeting human *NTRK1* (L-003159-00-0005) and ON-TARGET plus nontargeting siRNA (D-001810-0X) were purchased from Dharmacon (Horizon, US). SiRNA duplexes were transfected using the X-tremeGENE siRNA Transfection Reagent (cat. no.: 04476093001, Roche, Germany) according to the manufacturer’s instructions. Briefly, cells were transfected with siRNA duplexes at a final concentration of 20 nM. Seventy-two hours after siRNA transfection, the cells were either harvested for immunoblotting or used for cell proliferation or migration assays.

The ON-TARGET plus SMARTpool siRNA-*NTRK1* target sequence was as follows:

*NTRK1* (J-003159-09): 5’-GAGAGCAUCCUGUACCGUA-3’;
*NTRK1* (J-003159-10): 5’-ACACGCAACUGUCUAGUGG-3’;
*NTRK1* (J-003159-11): 5’-GGACAACCCUUUCGAGUUC-3’;
*NTRK1* (J-003159-12): 5’-CAACAAAUGUGGACGGAGA-3’;
Negative control: 5’-UUCUCCGAACGUGUCACGUTT-3’.

### Cell Proliferation and Colony Formation Assays

Cells were seeded at 2 × 10^4^ cells/ml in MEM/EBSS, supplemented with 10% FBS. The cells were treated with Ro 08-2750 (5 and 10 μM, cat. no.: 2272, TOCRIS, America), dimethyl sulfoxide (DMSO), Verteporfin (1 μM, VP, a YAP inhibitor, catalog no.: HY-B0146, MedChemExpress, China), and 200 ng/ml β-NGF (cat. no.: 450-01, PEPROTECH, US) for 24, 48, and 72 h. After treatment, cell numbers were counted using a hemocytometer. To carry out the colony formation assay, 200 cells/well were plated into 60-mm cell culture dishes, and colonies were counted after 2 or 3 weeks; the cells were fixed with methanol (≥ 99.5%) at room temperature for 15 min and stained with a 1% crystal violet solution at room temperature for 10 min. The NGF inhibitor was added to the cells every week. Only colonies containing more than 50 cells were regarded as positive colonies and counted. All experiments were conducted in triplicate.

### Wound Healing Assay

Confluent cells in 6-well plates were scratched using a 200 μl pipette tip and grown for 24 and 48 h in MEM/EBSS containing 10% FBS or medium without serum (for NGF treatment experiments). The scratch was imaged under a light microscope (magnification, × 100), and wound closure was assessed at different time points (0, 24, and 48 h). The widths of the scratches were analyzed with Image J (National Institutes of Health), and three biologically independent experiments were conducted.

### Western Blot Analysis and Antibodies

Cell lysates (total protein) were collected using RIPA lysis buffer, and the protein concentration was detected with a BCA kit (Proandy, China). According to the manufacturer’s instructions, cell or tumor tissue lysates were separated by 8–12% Bis-Tris Gels, under 72 V electrophoresis for 40 min, followed by 90 V electrophoresis for 90 min. After electrophoresis, proteins were transferred to NC membranes (PALL, Germany) under 320 mA for 100 min. After blocking with 5% non-fat milk or 5% BSA in TBS with 0.1% Tween-20 for 90 min at room temperature, the membranes were incubated with primary antibody overnight at 4°C. The next day, the membrane was then washed with Tris-Buffered Saline and Tween (TBST) for 30 min; the membranes were incubated with HRP-labeled secondary antibodies (dilution 1:3,000, cat. no.: ZB-2301, ZSGB-BIO, China) for 1.5 h, then the membrane was washed three times with TBST; chemiluminescence detection reagent was used to develop the Chemiluminescent Imager (Tanon-5200). Gel image system was used to analyze the band density (Bio-Rad Laboratories, Inc). All protein expression levels were normalized to the level of the internal standard control GAPDH (dilution 1:5,000, cat. no.: AP0063, Bioworld Technology, USA). The following antibodies were used for immunoblotting: anti-MST1 (dilution 1:1,000, cat. no.: 22245-1-AP), anti-LATS1 (dilution 1:1,500, cat. no.: 17049-1-AP) from Proteintech Group (Proteintech, USA); anti-Phospho-MST1 (Thr183)/MST2 (Thr180) (E7U1D) (dilution 1:1,000, cat. no.: #49332) antibody and anti-YAP (D8H1X) (dilution 1:1,000, cat. no.: #14074), anti-TrkA (dilution 1:1,000, cat. no.: #2505), anti-Phospho-YAP (Ser127) (D9W2I) (dilution 1:1,000, cat. no.: #13008), anti-Phospho-LATS1 (Ser909) (dilution 1:1,000, cat. no.: #9157) antibodies from Cell Signaling Technologies (Beverly, MA); and anti-NGF (dilution 1:800, cat. no.: Ab52918) from Abcam (England).

### Quantitative Real-Time PCR (qPCR)

Total RNA was extracted using TRIzol reagent (Invitrogen, USA), and only highly pure RNAs (1.7 < A260/A280 < 2.2) were used. RNA (1 µg) was converted into cDNA using the PrimeScript™ RT reagent Kit with gDNA Eraser (Takara, Japan). After 10-fold dilution, 4 µl of cDNA was subjected to PCR amplification using TB Green^®^ Premix Ex Taq™ II (Takara) according to the manufacturer’s protocol in a Bio-Rad CFX Manager. The following thermocycling conditions were used for qPCR: 95°C for 30 s, then 30 cycles with 95°C for 5 s, 60°C for 30 s, and 72°C for 40 s. Glyceraldehyde-3-phosphate dehydrogenase (GAPDH) was used as an internal control. The expressions of genes were quantified using the 2^-ΔΔCq^ method. The primer sequences were as follows:

ANKRD1-F: 5’-GCCAAAGACAGAGAAGGAGATAC-3’;
ANKRD1-R: 5’-GAGATCCGCGCCATACATAAT-3’;
CTGF-F: 5’-GGAAATGCTGCGAGGAGTGG-3’;
CTGF-R: 5’-GAACAGGCGCTCCACTCTGTG-3’;
GAPDH-F: 5’-GTGAAGGTCGGAGTCAACGG-3’;
GAPDH-R: 5’-GAGGTCAATGAAGGGGTCATTG-3’.

### Transwell Assay

Transwell assays were carried out using 24-well plates cell culture inserts (Corning, USA). The upper surface of 6.5-mm diameter filters with 8.0 μm pore. About 0.8 × 10^5^ viable cells in serum-free medium were seeded onto the upper chamber of each insert; complete medium was added to the bottom chamber. Following 24 h of incubation, treatment with DMSO, Ro 08-2750 (10 μM), β-NGF (200 ng/ml), or Verteporfin (1 μM), cells were washed twice with sterile 1 × PBS to remove the dead cells, and then the cells were fixed with methanol (≥ 99.5%) at room temperature for 15 min and stained with a 1% crystal violet (Solarbio, China) solution at room temperature for 15 min.

### Tumor Xenograft Assay

Four-week-old female nude mice (BALB/c) were purchased from Shanghai SLAC Laboratory Animal Co., Ltd. (Shanghai, China). The mice were divided randomly. The mice were bred in a specific pathogen-free condition in which the temperature is at 22–25°C and the humidity is at 40–50%. The cells in the logarithmic growth phase were counted under sterile conditions. HeLa cells (1 × 10^6^) were suspended in 100 μl phosphate-buffered-saline (PBS) and then injected subcutaneously into the back of the forelimbs of BALB/cnude mice. On day 10 after the implantation of tumor cells (when tumor volume reached ~50 mm^3^), tumor-bearing mice were randomly divided into four groups (*n =* 4 per group) and given different treatment. The tumor dimensions were measured every 2 days *via* digital caliper measurements. After 2 weeks, the mice were photographed and the tumors were removed and then sacrificed by cervical dislocation. The tumor volume was calculated with the formula V = (length × width^2^)/2. The experiment was approved by the Animal Ethics Committee of Xi’an Jiaotong University.

Human beta-nerve growth factor (β-NGF) (Peprotech, cat. no.: #450-01, America) stock solution at a concentration of 100 μg/ml was prepared in sterile water, and then the solution at a concentration of 10 μg/ml was stored in PBS containing 5% Trehalose. When tumor volume reached ~50 mm^3^ (day 10 after the implantation of tumor cells), β-NGF (1,440 ng/day for 14 days) was administered subcutaneously three times a day for 14 days (23).

Verteporfin (VP) (MedChemExpress, cat. no.: HY-B0146, China) was dissolved in DMSO at a concentration of 75 mg/ml, and then the solution at a concentration of 7.5 mg/ml was stored in 10% DMSO + 40% PEG 300 + 5% Tween-80 + 45% saline. In an *in vitro* study, cervical cells were treated with 1 μM verteporfin or DMSO for 24, 48, or 72 h at 37°C, 5% CO_2_, respectively, under the condition of darkness during both treatment and lysis. In an *in vivo* study, female nude mice were administered intraperitoneally (IP) at a dose of 100 mg/kg every 2 days for a total of 2 weeks (24); the control female nude mice were administered with equivalent PBS.

Mice were treated with Ro 08-2750 (TOCRIS, cat. no.: 2272, America) at 13.75 mg/kg body weight intraperitoneally, the highest dose achievable due to limited compound solubility and the use of DMSO as an excipient (25). Drug administration (Ro 08-2750, 13.75 mg/kg, DMSO) was performed by intraperitoneal injections after dividing the groups for pharmacodynamic experiments every 2 days for 2 weeks.

### Statistical Analysis

The statistical analyses were carried out using the GraphPad Prism software. All experiments were performed in triplicate, and all results are presented as mean ± SD. Analysis of differences between the two groups was performed using Student’s t test, one-way ANOVA. Significance is indicated as follows: *, P < 0.05; **, P < 0.01.

## Results

### NGF Induces Cervical Cancer Cells Proliferation and Migration Through Activating the Hippo Signaling Pathway

**Figure 1:**
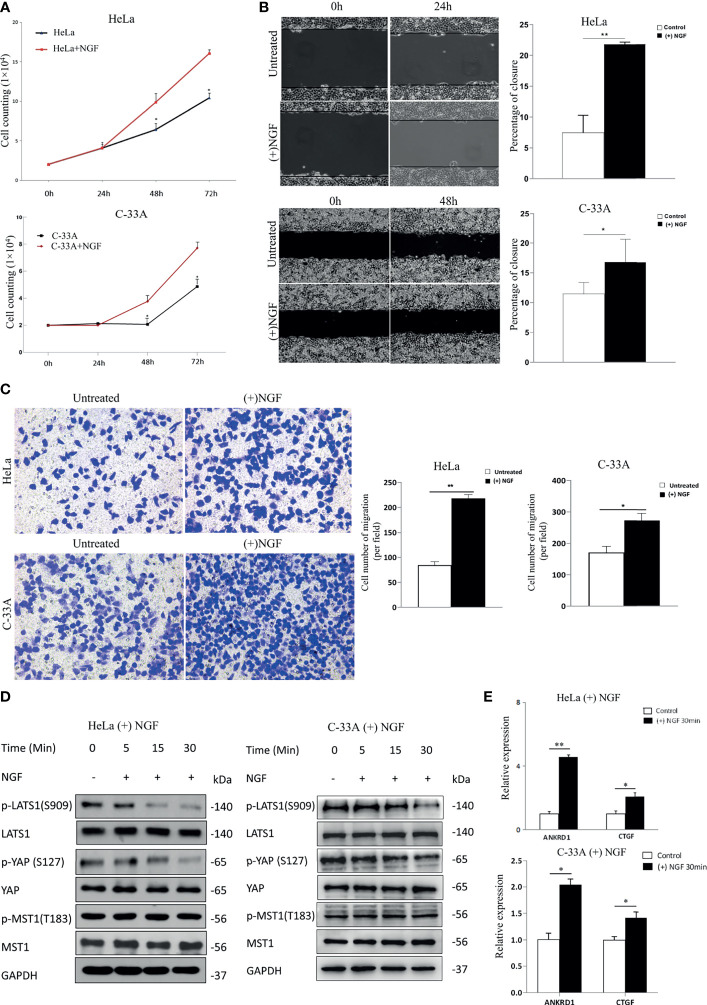
NGF induces cervical cancer cell proliferation and migration through activating the Hippo signaling pathway. **(A)** A growth assay was performed on standard culture plastic for various periods of time. Cells were treated with 200 ng/ml β-NGF every 24 h for 72 h. **(B)** Representative photographs showing a wound healing assay in the presence of NGF in serum-free medium for 24 h (left panel). Quantification of wound closure estimated from three independent experiments (right panel). **(C)** Representative photographs showing transwell assays in the presence of NGF in serum-free medium for 24 h (left panel). Quantification of the cell number of migration (per field) estimated from three independent experiments (right panel). **(D)** The samples were HeLa or C-33A cell lysates maintained in serum-free medium for 24 h with or without 200 ng/ml β-NGF treatment for 5, 15, or 30 min. GAPDH was used as a loading control. **(E)** Total RNA expression (qRT-PCR) of YAP target genes in HeLa cells treated without or with 200 ng/ml β-NGF for 30 min. Error bars represent SDs; *P < 0.05; **P < 0.01.

To explore the biological function of NGF in cervical cancer cells, we investigated the effect of NGF on HeLa and C-33A cell lines at certain concentration (200 ng/ml) (**Figure 1**). Cervical cancer cells were maintained in serum-free medium for 24 h prior to treatment with NGF (26). As shown in **Figure 1A**, exogenous β-NGF (200 ng/ml) efficiently stimulated cell proliferation and migration of two cervical cell lines in a dose-dependent manner. The effect of NGF on cell migration was examined by wound healing assay and Transwell assay. We found that the wound gaps in the untreated group were significantly wider than those in the NGF-treated groups (**Figure 1B**). Similar to the results of the wound healing assay, NGF effectively induced cell migration (**Figure 1C**).

MST and LATS are core kinase cascades of the Hippo pathway, while YAP and TAZ are downstream effectors. When Hippo signaling is activated, MST phosphorylates and activates LATS in mammalian cells. Then, activated LATS phosphorylates YAP at S127, providing a docking site for 14-3-3 proteins, sequestering YAP in the cytoplasm (27). In contrast, unphosphorylated YAP translocates into the nucleus, where it acts as a transcriptional coactivator to induce the expression of genes that promote cell proliferation and inhibit apoptosis. Therefore, to determine the relationship between NGF and the Hippo signaling pathway, we measured the protein levels of phospho-MST1 (p-MST1), p-LATS1 (the active form), and p-YAP (the inactive form) by western blot analysis. Our results showed that the levels of p-LATS1 and p-YAP in HeLa and C-33A cells treated with NGF were decreased in a time-dependent manner (**Figure 1D**). Using qRT-PCR, we found that the total RNA levels of the YAP target genes ANKRD1 and CTGF were also increased after NGF treatment (**Figure 1E**).

### Inhibition of NGF Suppresses the Proliferation and Migration of Cervical Cancer Cells Effectively

**Figure 2:**
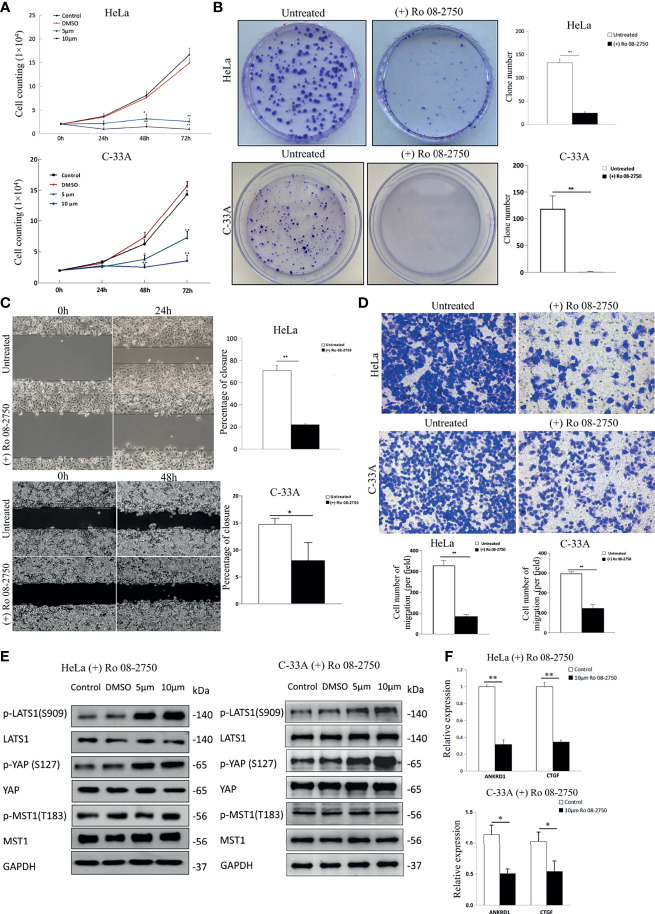
Inhibition of NGF suppresses the proliferation and migration of cervical cancer cells effectively. **(A)** The numbers of HeLa and C-33A cells were treated with DMSO, Ro 08-2750 (5 μM or 10 μM) in media containing 10% FBS; the numbers of cells every 24 h for 72 h were counted. **(B)** Representative images of a colony formation assay of HeLa and C-33A cells treated with or without 10 μM Ro 08-2750 for 3 weeks (left panel). The results of three independent colony formation assay experiments were quantified (right panel). **(C)** Representative images of a wound healing assay of cervical cancer cell lines treated with or without 10 μM Ro 08-2750 for 24 h (HeLa cells) or 48 h (C-33A cells) and incubated in medium containing 10% serum (left panel). The extent of wound closure estimated from three independent experiments was quantified (right panel). **(D)** Representative images of transwell assays of cervical cancer cell lines treated with or without 10 μM Ro 08-2750 for 24 h and incubated in medium containing 10% serum (top panel). The extent of the cell number of migration (per field) estimated from three independent experiments was quantified (bottom panel). **(E)** Representative Western blot analysis of lysate from HeLa and C-33A cells treated with solvent (DMSO, 24 h) or Ro 08-2750 (5 or 10 μM, 24h). GAPDH was used as a loading control. **(F)** Total RNA expression of YAP target genes in cervical cancer cell lines treated with or without 10 μM Ro 08-2750. All results are the mean of at least three independent experiments, with each performed in triplicate. Error bars represent SDs; *P < 0.05; **P < 0.01.

The results presented thus far indicate that NGF stimulation inactivates LATS1 and activates YAP. To test the converse hypothesis, whether the NGF inhibitor activates LATS1 and inactivates YAP function, we first treated HeLa and C-33A cells with Ro 08-2750, an NGF inhibitor that can block the binding of NGF to TrkA and p75^NTR^ (28). Cell count assay was used to assess the effect of Ro 08-2750 in cervical cancer cell proliferation. After 24, 48, and 72 h of incubation with Ro 08-2750 (5 and 10 μM), the cellular proliferative activity was significantly lower in a dose- and time-dependent manner compared to those in the control group and the DMSO group (**Figure 2A**).

The plate colony formation assay reflects anchorage-independent growth potential and proliferation. Ro 08-2750 also significantly decreased the numbers and sizes of the colonies (**Figure 2B**). Next, we assessed the migratory potential of HeLa and C-33A cells treated with Ro 08-2750. According to the wound healing assay results, cells in the control group almost filled the gap within 24 h (HeLa cells) or 48 h (C-33A cells), whereas HeLa and C-33A cells in the Ro 08-2750-treated groups failed to migrate into the wound (**Figure 2C**). Cell migration was assessed with transwell assay after being Ro 08-2750-treated for 24 h, and similar to the results of the wound healing assay, the NGF inhibitor effectively reduced cell migration (**Figure 2D**).

To test whether the NGF inhibitor activates the Hippo pathway, we measured the active forms of p-MST1 and p-LATS1, and the inactive form of p-YAP (S127). As shown in **Figure 2E**, we found that although Ro 08-2750 treatment induced LATS1 and YAP1 phosphorylation, there were no notable effects on MST1 phosphorylation. In addition, the expression of several YAP target genes (CTGF, ANKRD1) was suppressed by Ro 08-2750 treatment (**Figure 2F**). Taken together, these results suggest that the NGF inhibitor activates LATS1 and suppresses YAP transcriptional coactivator function.

### Knockdown of TrkA Inhibits Cervical Cancer Cells Proliferation and Migration on the Hippo Pathway

**Figure 3:**
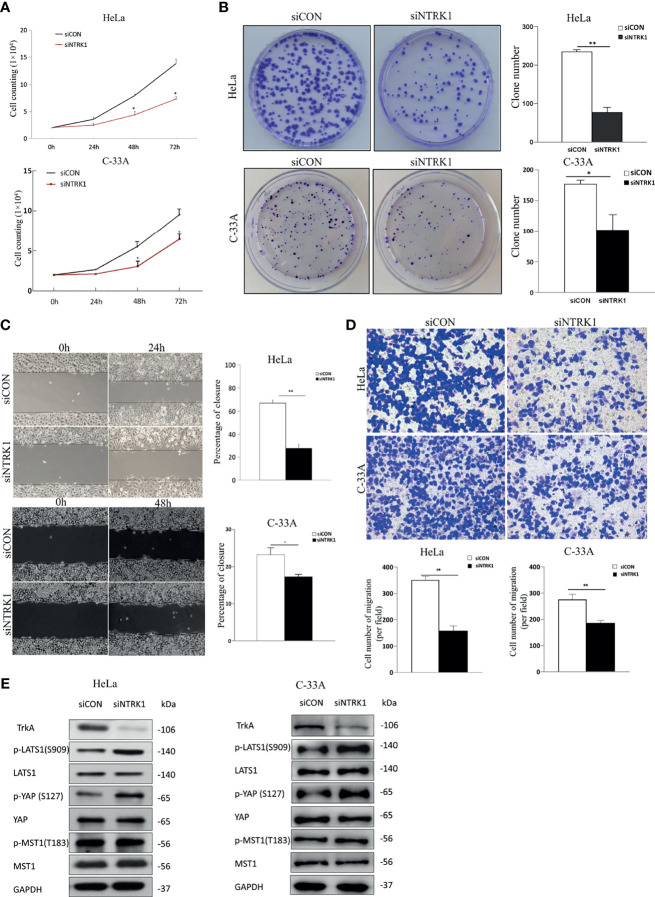
Knockdown of TrkA suppresses cervical cancer cell line proliferation and migration. **(A)** The numbers of siCON or si*NTRK1*-transfected HeLa and C-33A cells were counted every 24 h for 72 h. **(B)** Representative images of a colony formation assay with siCON or si*NTRK1*-transfected HeLa and C-33A cells after 3 weeks (left panel). The results of three independent colony formation assay experiments were quantified (right panel). **(C)** Representative images of a 24 h (HeLa cells) or 48 h (C-33A cells) wound healing assay with siCON or si*NTRK1*-transfected cervical cancer cell lines (left panel). The extent of wound closure estimated from three independent experiments (right panel). **(D)** Representative images of transwell assays of cervical cancer cell lines treated with siCON or si*NTRK1*-transfected cervical cancer cell lines (top panel). The extent of cell number of migration (per field) estimated from three independent experiments was quantified (bottom panel). **(E)** Western blot analysis of lysates from siCON or siNTRK1-transfected HeLa and C-33A cells was performed with anti-TrkA, anti-LATS1, anti-p-LATS1, anti-YAP, anti-p-YAP (S127), anti-MST1, and anti-p-MST1 (T183) antibodies. GAPDH was used as a loading control. Error bars represent SDs; *P < 0.05, **P < 0.01.

To further explore the functional role of NGF in cervical cancer cells, small interfering RNAs (siRNAs) were used in cervical cancer cells to specifically knockdown TrKA expression. TrkA knockdown (si*NTRK1*) significantly inhibited cell proliferation, migration, and invasion of cervical cells, as measured by cell proliferation, colony formation assays, wound healing assay, and transwell assay (**Figures 3A–D**). Additionally, TrkA knockdown increased the levels of p-LATS1 (the active form of LATS1) and p-YAP-S127 (the inactive form of YAP) (**Figure 3E**).

### Verteporfin Eliminates the Effect of NGF Through Hippo Pathway

Verteporfin (VP) is a suppressor of the YAP1-TEAD complex (29), also known as an FDA-approved drug used as a photosensitizer for photodynamic therapy in patients with age-related macular degeneration (30).

**Figure 4:**
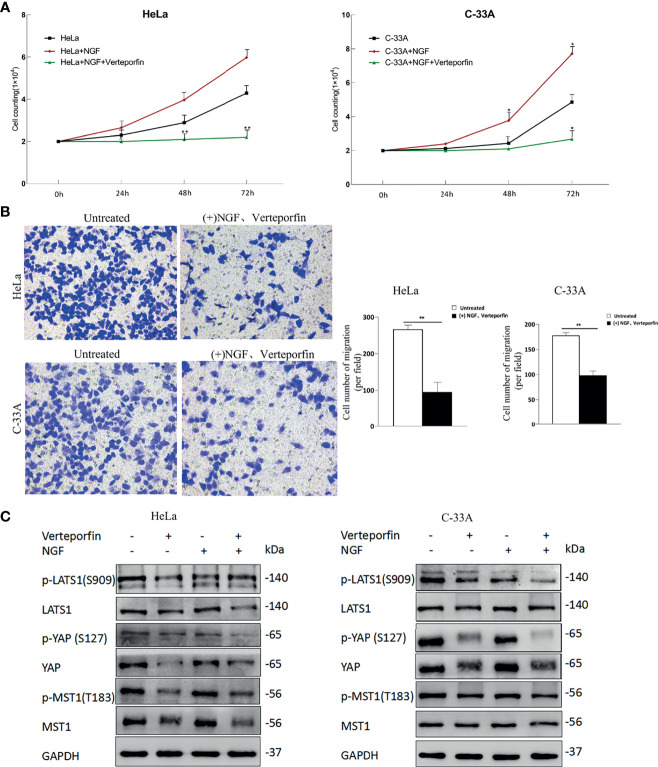
Verteporfin eliminates the effect of NGF through the Hippo pathway *in vitro*. **(A)** The numbers of HeLa and C-33A cells were treated with 200 ng/ml β-NGF and Verteporfin (1 μM); the numbers of cells every 24 h for 72 h were counted. **(B)** Representative photographs showing transwell assays in the presence of NGF in serum-free medium for 24 h with Verteporfin (1 μM) (left panel). Quantification of cell number of migration (per field) estimated from three independent experiments (right panel). Magnification: 200 ×. **(C)** The samples were HeLa or C-33A cell lysates maintained in serum-free medium for 24 h with or without 200 ng/ml β-NGF treatment and Verteporfin (1 μM). GAPDH was used as a loading control. Data are shown as the mean ± SD in three independent experiments. *P < 0.05, **P < 0.01.

After initial evaluation of the biological effects of NGF *via* the Hippo pathway, we studied the *in vitro* effects of verteporfin. Cells were treated with 200 ng/ml β-NGF together with 1 µM verteporfin every 24 h for 72 h to determine whether VP affects NGF through active YAP. As shown in **Figure 4A**, the stimulating effect of NGF on cell proliferation was completely reversed by VP. Increases in migration mediated by NGF were also reversed after VP treatment according to transwell assays (**Figure 4B**). Interestingly, we examined the protein expression of the Hippo pathway in cervical cell lines treated with control, Verteporfin, NGF, and NGF with Verteporfin groups by western blot. Verteporfin and NGF with Verteporfin groups reduced Hippo pathway protein levels (p-LATS1, LATS1, p-YAP, YAP, p-MST1, MST1) in both HeLa and C-33A cell lines, compared to the normal control group (**Figure 4C**). Thus, verteporfin decreases cervical cell Hippo/YAP signaling pathway and increases apoptosis.

### NGF Promoted Cervical Cancer Cell-Derived Xenograft Tumors in Nude Mice by Inhibiting the Hippo Pathway

**Figure 5:**
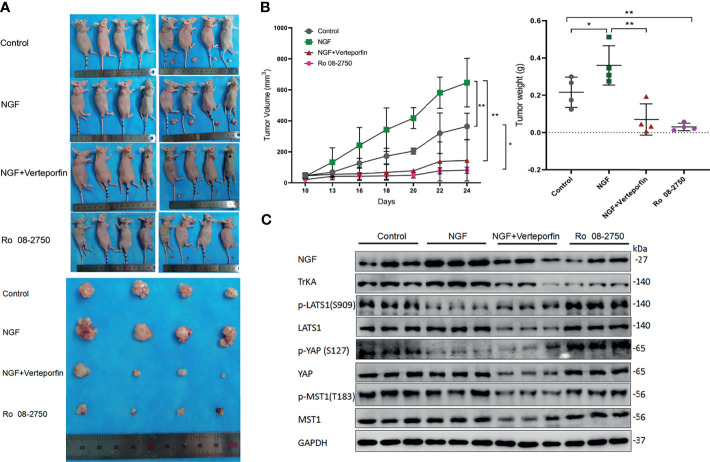
NGF promoted cervical cancer cell-derived xenograft tumors in nude mice by inhibiting the Hippo pathway. **(A)** HeLa cells were injected into the female nude mice (n = 16); Control group: PBS were injected into the nude mice; NGF group: β-NGF (1,440 ng/days for 14 days) was administered subcutaneously three times a day for 14 days; NGF with Verteporfin group: β-NGF (1,440 ng/day for 14 days) was administered subcutaneously three times a day with verteporfin administered intraperitoneally at a dose of 100 mg/kg every 2 days for a total of 2 weeks; Ro 08-2750 group: Ro 08-2750 (13.75 mg/kg) was injected intraperitoneally into the nude mice. Representative images of xenograft tumors are shown. **(B)** Tumor volume and weight in the xenograft mice from Control, NGF, NGF with Verteporfin, and Ro 08-2750 groups. Data are shown as the mean ± SD in three independent experiments. *P < 0.05, **P < 0.01. **(C)** Western blot analysis of lysates from xenograft tumors in nude mice was performed with anti-NGF, anti-TrkA, anti-p-LATS1, anti-LATS1, anti-p-YAP, anti-YAP, anti-p-MST1, and anti-MST1 antibodies. GAPDH was used as a loading control.

In order to verify the effect of NGF on cervical tumor growth and whether it correlates with the Hippo pathway, the xenograft mouse model was generated by subcutaneous injection of HeLa cell line. No animals died during the test. The mass of tumor could be felt by touch on day 10 after the implantation of tumor cells (when tumor volume reached ~50 mm^3^), tumor-bearing mice were randomly divided into four groups (n = 4 per group) and given different treatment.. Six days after being given different treatment, tumor size increased gradually in the Control group and the NGF group, but it grew more slowly in the NGF with Verteporfin group as well as in the Ro 08-2750 group. After treatment for 6 days, tumor size in the NGF with Verteporfin group and the Ro 08-2750 group began to decrease, while it kept increasing in the Control group and the NGF group. The tumor size showed significant difference among different groups after 10 days (P < 0.05). By observing tumor volume and weight in the nude mice model, it was found that NGF subcutaneous injection significantly promoted the formation of subcutaneous tumors of cervical cancer cells. The Ro 08-2750 group could reduce the tumor formation compared to the normal controls, while NGF with verteporfin injection abrogated the NGF-enhanced tumorigenicity *in vivo* (**Figures 5A, B**).

Next, to determine whether NGF affects the Hippo pathway, immunoblots were carried out on tumors of different groups. As expected, when these tumors were treated with NGF, we observed a decrease in p-YAP and p-LATS1 protein levels. In contrast, protein levels had significantly higher p-LATS1 and p-YAP in the Ro 08-2750 group. Likewise, when these tumors were treated with NGF and Verteporfin, the previously observed decrease in p-YAP and p-LATS1 protein levels was eliminated, further corroborating the critical role of NGF signaling for the Hippo pathway (**Figure 5C**).

## Discussion

YAP is the major downstream effector of the Hippo pathway. Activation of the Hippo pathway results in the phosphorylation and sequestration of YAP into the cytoplasm, which deactivates YAP-regulated gene transcription (31). Altering YAP function represents a potential therapeutic intervention for tumors. Importantly, the Hippo pathway is regulated by a network of upstream components and mechanisms rather than extracellular signaling peptides and receptors. Since all upstream regulators affect YAP nuclear transcriptional responses and availability, inhibiting YAP translocation into the nucleus is theoretically a valid way to suppress its oncogenic function, regardless of the dependency of YAP on the Hippo signaling cascade (32, 33).

In this study, the stimulation of NGF led to cervical cancer cell proliferation and migration and promoted the transcription of YAP target genes. The NGF inhibitor Ro 08-2750 suppressed the proliferation and migration of HeLa and C-33A cells. Notably, the levels of p-LATS1 and p-YAP treated with NGF were decreased in a time-dependent manner in HeLa and C-33A cells. On the other hand, NGF inhibitor treatment induced LATS1 and YAP1 phosphorylation and also suppressed the transcription of YAP target genes, suggesting crosstalk between NGF and YAP. Consistent with these results, knockdown NGF inhibitor by siRNA suppressed cell proliferation and migration and increased the expression of active p-LATS1 and inactive YAP (p-YAP-S127). It is interesting that verteporfin, which inhibits YAP1/TEAD interaction, eliminates the effect of NGF through the Hippo pathway; a common theme is NGF with verteporfin suppresses cell proliferation and migration as well as reduces the level of p- LATS1, LATS1, p-YAP, YAP, p-MST, and MST (34). These results are confirmed in the xenograft tumor mice. What is more, the expression level of TrKA increased in the NGF group compared to that in the control group, whereas those in the NGF with Verteporfin group as well as the Ro 08-2750 group were decreased significantly, indicating that Verteporfin and Ro 08-2750 could inhibit the expression of TrKA receptors *in vivo* while the protein expression level of NGF is similar among Control, NGF with Verteporfin, and Ro 08-2750 groups. Moreover, it is interesting that C-33A cells have a significant difference after 48 h, which means the growth rate and the ability to respond to drugs of C-33A cells are not as good as HeLa cells. We speculate that the differential proliferation effect could be that Hela cells and C-33A have different HPV infection status. For example, Benjamin Kansy investigated the expression patterns of CD 44 and AREG, two signaling molecules essential for cell proliferation and differentiation, under the influence of selective TKIs in HPV+ and HPV- squamous carcinoma cell lines. The expression of AREG and CD44 was dependent on the cell line’s HPV status (35).

**Figure 6:**
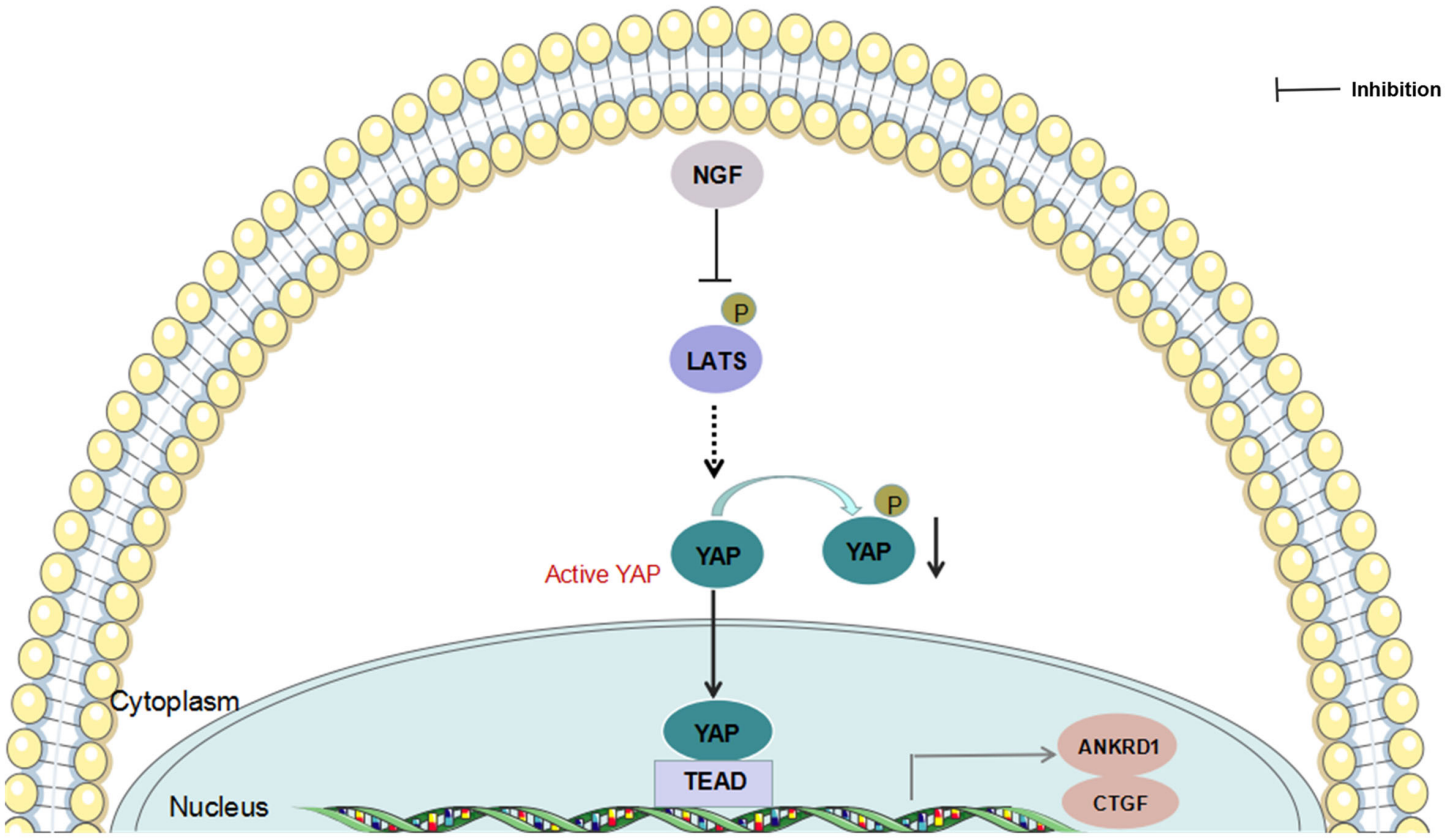
Schematic presentation of NGF-induced regulation of YAP function.

Taken together, our findings provide a potential mechanism for NGF inhibition-mediated YAP suppression (**Figure 6**). Understanding how NGF alters Hippo pathway protein interactions and their posttranslational modifications to activate downstream signaling events is important. As in proliferating cells, YAP coactivates TEAD transcription factors, leading to the expression of survival genes involved in the suppression of proliferation and apoptosis when the Hippo pathway is inactive. In contrast, active YAP promotes cancer cell migration and invasion. However, inhibition of NGF stimulation posttranslationally modulates LATS1/2 phosphorylation and YAP expression. Our data expand the understanding of NGF signaling actions with the Hippo signaling pathway. NGF may function as a mediator of cervical cancer progression by modulating Hippo/YAP pathway. Although more work is needed to fully elaborate the mechanism of cervical cancer progression mediated by NGF through Hippo/YAP pathway, the results presented here may provide some new therapeutic opportunities that anchor to the interaction between NGF signaling and Hippo/YAP pathway in cervical cancer.

## Data Availability Statement

The original contributions presented in the study are included in the article material. Further inquiries can be directed to the corresponding authors.

## Ethics Statement

This study was approved by the Ethics Committee of the First Affiliated Hospital of Xi’an Jiaotong University.

## Author Contributions

LW: Cell experiment (C-33A cells), xenograft tumors in nude mice, conceptualization, methodology, software, formal analysis, investigation, data curation, writing (original draft preparation), and writing (review and editing). JL: validation and cell experiment (HeLa cells). RoW: validation and data curation. HC: formal analysis. RuW: formal analysis. XY and WW: cell experiment (HeLa cells), conceptualization, validation, resources, writing (review and editing), visualization, supervision, project administration, and funding acquisition. All authors contributed to the article and approved the submitted version.

## Funding

This work was supported by the fund of “Project A of the First Affiliated Hospital of Xi’an Jiaotong University (XJTU-2021-01).”

## Conflict of Interest

The authors declare that the research was conducted in the absence of any commercial or financial relationships that could be construed as a potential conflict of interest.

## Publisher’s Note

All claims expressed in this article are solely those of the authors and do not necessarily represent those of their affiliated organizations, or those of the publisher, the editors and the reviewers. Any product that may be evaluated in this article, or claim that may be made by its manufacturer, is not guaranteed or endorsed by the publisher.

## Abbreviations

ANKRD1, Ankyrin repeat domain 1; CTGF, connective tissue growth factor; CYR61, cysteine-rich angiogenic inducer 61; DMSO, dimethyl sulfoxide; IP, intraperitoneal; GAPDH, glyceraldehyde-3-phosphate dehydrogenase; LATS1, large tumor suppressor 1; MST1, mammalian Ste20-like protein kinases 1; PBS, phosphate-buffered saline; qRT-PCR, Quantitative real-time polymerase chain reaction; siRNA, Small interfering RNA; TAZ, transcriptional coactivator with a PDZ-binding domain; VP, verteporfin; WB, Western blotting; YAP, Yes-associated protein; p-YAP, phospho-YAP.
